# Training of workplace-based clinical trainers in family medicine, South Africa: Before-and-after evaluation

**DOI:** 10.4102/phcfm.v10i1.1589

**Published:** 2018-05-31

**Authors:** Robert Mash, Julia Blitz, Jill Edwards, Steve Mowle

**Affiliations:** 1 Division of Family Medicine and Primary Care, Stellenbosch University; 2 Royal College of General Practitioners, London, United Kingdom

## Abstract

**Background:**

The training of family physicians is a relatively new phenomenon in the district health services of South Africa. There are concerns about the quality of clinical training and the low pass rate in the national examination.

**Aim:**

To assess the effect of a five-day course to train clinical trainers in family medicine on the participants’ subsequent capability in the workplace.

**Setting:**

Family physician clinical trainers from training programmes mainly in South Africa, but also from Ghana, Uganda, Kenya, Malawi and Botswana.

**Methods:**

A before-and-after study using self-reported change at 6 weeks (*N* = 18) and a 360-degree evaluation of clinical trainers by trainees after 3 months (*N* = 33). Quantitative data were analysed using the Statistical Package for Social Sciences, and qualitative data were analysed thematically.

**Results:**

Significant change (*p* < 0.05) was found at 6 weeks in terms of ensuring safe and effective patient care through training, establishing and maintaining an environment for learning, teaching and facilitating learning, enhancing learning through assessment, and supporting and monitoring educational progress. Family physicians reported that they were better at giving feedback, more aware of different learning styles, more facilitative and less authoritarian in their educational approach, more reflective and critical of their educational capabilities and more aware of principles in assessment. Despite this, the trainees did not report any noticeable change in the trainers’ capability after 3 months.

**Conclusion:**

The results support a short-term improvement in the capability of clinical trainers following the course. This change needs to be supported by ongoing formative assessment and supportive visits, which are reported on elsewhere.

## Introduction

South Africa is on a journey towards improving universal health coverage through national health insurance, which requires the revitalisation of primary health care (PHC) in the country.^1^ A number of initiatives have been implemented to try and improve the quality of PHC. One of these is strengthening PHC through the introduction of family physicians.^2^ Currently, there is no requirement for doctors working in PHC to have additional postgraduate training to equip them for work in this setting.

Effective PHC is delivered by multidisciplinary teams that usually include a family physician (FP).^3,4,5^ A FP is a specialist in family medicine who functions as an expert medical generalist within the PHC system.^2^ In the South African context, the FP often works at the district hospital as well as PHC and therefore may require an extended range of skills in surgery, obstetrics and anaesthetics. Most primary care consultations are with nurse practitioners, and many clinics have limited access to a doctor. Another initiative to strengthen PHC has been the introduction of community-oriented primary care with teams of community health workers providing services to designated groups of households.^1^ In this context, six roles for the FP have been identified:^2^ a competent clinician who can offer patient-centred holistic care in PHC and the district hospital, a consultant to the health care team who sees more complicated or difficult patients, a capacity builder who improves the competence of the health care team, a clinical trainer for students in formal education, a leader of clinical governance who improves the quality of services and a champion of community-orientated primary care who supports the development of community-based services.

Postgraduate training in family medicine has been formalised since 2007, and the first full-time training programmes were initiated in 2008, with graduates emerging since 2011.^2^ The South African human resources for health policy suggested that we needed to train 900 family physicians for the public sector in order to meet the immediate gap in supply.^6^ The national position paper on family medicine recommended an initial goal of 680 posts in the public sector to enable one FP to be placed at each sub-district, community health centre and district hospital across the country.^2^ Between 2011 and end of 2017, the nine training programmes took in 548 registrars and graduated 137 family physicians, with an average pass rate in the national examination of less than 50%. Although the reasons for this limited output are multifactorial, one of the key issues is the quality of clinical training. Postgraduate training of specialists in family medicine in the district health services is a new phenomenon for both the educational and health systems as such training for most other specialities predominantly takes place in referral hospitals.

Although the number of FPs on the district hospital platform has increased over the last few years and the numbers fulfil the Health Professions Council of South Africa requirements for the ratio of trainers to registrars, this does not necessarily imply that the FPs are capacitated to deliver quality clinical training. The capacity to do this may depend on contextual issues as well as the actual capability of the FP trainer. Contextual issues, for example, may include whether the FP is in a joint post with the university, whether the FPs have support of the facility and district managers, and whether their workload allows space for educational interactions.

In order to address the issue of capability, Stellenbosch University contextualised a five-day course on clinical training in the workplace developed by the Royal College of General Practitioners (RCGP) in the UK. The course was offered to FP trainers from all training programmes in the country.

Kirkpatrick describes four levels of outcomes that can be used to evaluate a course.^7^ At the simplest level, these involve gathering information on participants’ reaction or satisfaction. International literature on faculty development generally shows self-reported positive changes in knowledge and behaviour (Kirkpatrick Level 1).^8^ In wondering about the impact of this training, we were curious about the extent of actual teaching behaviour change (Kirkpatrick Level 3), but more particularly the impact on those being taught (Kirkpatrick Level 4a). This study aimed to assess the effect of the course on the participants’ capability to be a clinical trainer in the workplace.

## Methods

### Study design

A before-and-after study using self-reported change at 6 weeks and a 360-degree evaluation of clinical trainers after 3 months.

### Setting

The offering of the course was not planned primarily as a research project, but as an educational intervention with the purpose of improving the capability of clinical trainers in the workplace. The course was initially offered twice to the nine training programmes in South Africa during 2016, and in the final offering of the course in 2017, half the places were offered to clinical trainers from five other African countries (Ghana, Uganda, Kenya, Malawi and Botswana).

The majority of participants were FPs from South African training programmes where registrars were working within training complexes that consisted of community-based services, primary care facilities, district hospitals as well as regional or tertiary hospitals. Within the complexes, they were primarily trained by FPs on a ratio of up to four registrars to one FP (4:1). All programmes trained registrars over a four-year period and aligned their training with an agreed set of national learning outcomes and required clinical skills. All programmes used a single national exit examination offered by the College of Family Physicians that was usually sat during the fourth year of training.

FPs involved in the training of registrars could also be involved in the training of medical students, interns and sometimes clinical associates (mid-level doctors). Skills in clinical training would also be applicable to capacity building within the health care team, particularly with nurse practitioners and junior doctors.

The training programmes in other countries were also over four years and trained registrars in rural district hospitals (Kenya, Malawi, Botswana) or from a central referral hospital (Ghana, Uganda) and the associated primary care platform.

### Selection of participants

The Head of Department at each of the nine South African training programmes selected two FPs who were working as clinical trainers for the courses in February and August 2016. In February 2017, the course was offered to one FP from each of the South African training programmes and 10 FPs from other African countries (Ghana, Uganda, Kenya, Malawi, Botswana) who were also selected by their Head of Department. Altogether there were potentially 55 participants in this study.

### Training course

The five-day training course was facilitated by two experts from the RCGP as well as four locally trained facilitators. The aims of the course were to:

- promote the development of Family Medicine training programmes
- create and support a core group of clinical trainers familiar with contemporary methods of medical education
- initiate the development of a group of competent and confident educators capable of training family medicine registrars in the clinical setting
- provide opportunities for individual professional development through shared learning.

The course combined educational methods such as brief presentations, small group work, role play and simulated teaching practice, homework assignments and reflective learning. During the sessions, there was a focus on the importance of assessing the learning needs of the learners to support their professional development. The course introduced the concepts of teaching, learner-centred learning, assessment and curriculum. The programme addressed the following content:

- introduction: course goals and learning styles
- establishing and maintaining a learning environment
- national curriculum and learning outcomes
- facilitating reflective practice
- workplace-based assessment
- giving feedback
- teaching consultation skills
- micro-teaching
- registrars in difficulty
- leadership and strategic capacity

All participants were supplied with the course materials and a textbook on clinical training.^9^

### Data collection

A 360-degree survey tool was adapted from the previously validated Family Physician Impact Assessment Tool.^10^ The section of that tool that assesses the role of the FP as a supervisor or clinical trainer was selected, and additional statements formulated to evaluate specific competencies taught during the five-day course were added. The content and construct of the adapted tool were validated by the course facilitators who represented expertise in professional education, the course content and the South African context.

The 360-degree tool consists of 10 statements that related to the capability of the FP as a clinical trainer. The questionnaire was completed by the participant and by at least 5 (preferably 10) health workers in their workplace who had insight into the capability of the FP as a clinical trainer. These people were identified by the FP and could include registrars, interns, trainee clinical associates or medical students who they have a formal supervisory relationship with. In addition, other health workers that the FP had an educational relationship with in terms of capacity building could also be included.

Respondents completed a four-item Likert scale for each statement ranging from strongly disagree to strongly agree. They could also answer that this was not part of the FP’s work or that they were unable to express an opinion on this aspect of the FP’s training activities.

The questionnaire was completed at baseline with respondents evaluating the FPs’ performance for three months prior to the course. The questionnaire was completed again three months after the course. This before-and-after evaluation was completed for the two courses offered in 2016. Each time the FP disseminated the questionnaires to the eligible respondents. Respondents completed a questionnaire, placed it in a sealed envelope and posted it in a secure container that was available in a neutral location. The sealed container was then collected by courier. Ideally, the same respondents were asked to complete the questionnaire before and after the course, but this was not always practically possible.

Participants in the February 2017 course were asked to complete a self-reported questionnaire on their capability as a clinical trainer before and six weeks after the training course. The questionnaire was based on the 2014 version of the National Health Service’s Pan London Quality (NHSPLQ) and Regulation Unit’s General Practice Educator Application Form and the two-yearly educator self-reporting form. There were five sections:

- ensuring safe and effective patient care through training
- establishing and maintaining an environment for learning
- teaching and facilitating learning
- enhancing learning through assessment
- supporting and monitoring educational progress.

For each section, respondents were asked to score themselves on a scale from 1 (struggling to be effective) to 5 (excellent) in terms of their effectiveness as a clinical trainer. In addition, they were asked to provide written qualitative feedback on how their clinical training practice had been changed by the course. This qualitative data were collated and thematically analysed in order to provide more insight into any changes seen in the quantitative evaluation. The funder’s timeline excluded these participants from a 360-degree evaluation 3 months after the training.

### Data analysis

Quantitative data were captured in Excel and checked for errors or omissions. Data were entered into the Statistical Package for the Social Sciences Version 23 for further analysis. For the 360-degree tool, the average number of respondents per participant in the Training of Clinitcal Trainers (TCT) was 10 (range 5–11), and a mean score for each statement and for each participant was calculated from their respondent scores. This mean score for each statement per participant was then used in the further analysis. As the data were not normally distributed, it was further described using medians and interquartile ranges. Paired data were analysed using the Wilcoxon Signed Ranks test to assess statistically significant differences from before to after the course.

Qualitative data were analysed using an abbreviated framework method. After familiarisation with the data, the written feedback was collated into one chart and analysed thematically.

### Ethical considerations

Ethics approval was obtained from the University of Stellenbosch Health Research Ethics Committee (reference N11/09/278). Demographic details of the participants were not recorded in order to ensure their anonymity.

## Results

Altogether 33 FPs provided before-and-after quantitative 360-degree data from the two courses in 2016 and 18 FPs provided self-reported and qualitative data from the course in 2017. Table 1 compares the self-reported median scores before and 6 weeks after the training course and demonstrates a significant improvement across all five areas of effective clinical training.

**TABLE 1:** Self-reported before-and-after evaluation by clinical trainers at 6 weeks (*N* = 18).

Variable	Before median (IQR)	After median (IQR)	*p*
Safe and effective patient care	1.00 (1.0–2.0)	3.00 (2.0–3.0)	0.001
Environment for learning	1.00 (1.0–2.0)	2.50 (2.0–3.0)	< 0.001
Teaching and facilitating learning	1.00 (1.0–2.0)	2.50 (2.0–3.0)	0.001
Assessment	1.00 (1.0–2.0)	2.50 (2.0–3.0)	0.001
Educational progress	1.00 (1.0–2.0)	2.50 (2.0–3.0)	0.001

IQR, interquartile range.

Table 2 compares the 360-degree median scores before and 3 months after the training course and does not demonstrate any significant improvements.

**TABLE 2:** Before-and-after evaluation of clinical trainers by trainees (*N* = 33).

Criteria	Before median score (IQR)	After median score (IQR)	*p*
1. The family physician contributes to the training of interns or community service doctors.	3.7 (3.5–3.9)	3.7 (3.6–4.0)	0.284
2. The family physician contributes to the training of family medicine registrars.	3.7 (3.5–3.9)	3.8 (3.6–4.0)	0.205
3. The family physician contributes to the training of undergraduate students.	3.8 (3.6–3.9)	3.8 (3.5–4.0)	0.150
4. The family physician is involved in the assessment of under-graduate and/or postgraduate students	3.8 (3.6–3.8)	3.7 (3.5–3.9)	0.638
5. Having students supervised by the family physician has a positive impact on the quality of care at the facility, for example, through student projects.	3.7 (3.5–3.8)	3.7 (3.4–3.8)	0.162
6. The family physician gives meaningful, formative verbal feedback that assists with individual learning in the workplace	3.6 (3.4–3.7)	3.7 (3.6–3.8)	0.110
7. The family physician competently assesses clinical skills using assessment tools (such as the mini-CEX or DOPS)	3.4 (3.2–3.6)	3.6 (3.4–3.7)	0.231
8. The family physician is a role model of what is expected across all his or her other roles (e.g. clinician, consultant, clinical governance and capacity builder)	3.6 (3.4–3.8)	3.7 (3.6–3.8)	0.137
9. The family physician is supportive of the student’s resilience in the face of difficult personal or professional circumstances	3.5 (3.3–3.7)	3.5 (3.4–3.8)	0.412
10. The family physician creates a culture of learning in the workplace for all staff, not just students	3.6 (3.4–3.7)	3.6 (3.3–3.8)	0.732
11. The family physician uses a facilitative (rather than directive) style of training	3.6 (3.3–3.6)	3.6 (3.3–3.7)	0.158
12. The family physician is confident in their role as supervisor or trainer	3.6 (3.4–3.8)	3.7 (3.5–3.9)	0.231
13. Staff and students comment on how approachable the family physician is as a supervisor or trainer or mentor	3.4 (3.2–3.6)	3.5 (3.2–3.7)	0.149

IQR, interquartile range, CEX, Clinical Evaluation Exercise; DOPS, Direct Observation of Procedural Skills.

The strongest theme was around feedback to students and the use of a more structured approach with tools such as agenda-led outcome-based analysis (ALOBA) and Pendleton’s rules for giving feedback:

‘I have been more proactive in giving feedback. I have followed a structured process of giving feedback relying on the Pendelton’s method. I have discussed the method with the registrars and encourage them to use it among themselves and when giving feedback to medical students.’ (Participant 1, Male, Botswana)

Participants also found it useful to be more aware of their own preferred learning styles and that of their students:

‘Over the last six weeks as a result of attending the TCT course, I am aware of the different learning styles of the registrars. This guides me to direct my teaching efforts to the individual learning needs of individual registrars.’ (Participant 2, Male, Uganda)

Participants reported a conscious shift in their style of teaching to be more student-centred and facilitative:

‘I have certainly been more aware of how I can encourage my students to recognise their learning styles, then to facilitate their learning around this. I am also aware of my own blind spots with regards to teaching and how I was assessing my students, this has helped me to start thinking out of the box more, thus encouraging more student interaction during tutorials, and really playing more of facilitation role. I have been giving much more feedback to my students.’ (Participant 3, Male, South Africa)

Tools for reflection on their own training and teaching were useful. There was more awareness of their need for development as a teacher and of the educational issues as a whole:

‘I am more critical of myself after the teaching. I had a false sense of security before and I would feel proud of my teaching. Whereas now I know more and I know what I should be working on, I am more critical and reflect more than I used to.’ (Participant 4, Female, South Africa)

Participants spent more time planning their teaching and training as well as helping students to plan their own learning:

‘I really understand the place of the portfolio better and therefore the planning for a rotation with me is more specific and directed.’ (Participant 5, Male, South Africa)

The course provided participants with more insight into the principles of assessment. This included the need to link assessment to curriculum outcomes and learning opportunities. It also included more awareness of the need to assess the validity and reliability of assessment:

‘Thinking through the ‘final product/outcome – assessment purpose – teaching and learning’ process when considering proposed learning interventions.’ (Participant 6, Female, Malawi)

‘I am very cognisant of the reliability and validity of each and every assessment method I use with the registrars.’ (Participant 2, Male, Uganda)

The textbook was found to be a useful resource and reinforced the course content:

‘I am reading more about clinical teaching (I was given a book at the TCT which I am finding very useful and appropriate). I think the book is helpful because it follows the course and what we were taught on the TCT course. So when I need to recap something, it is there, almost the same as how it was presented (along the same lines) – which really helps to consolidate my learning. And I can read further on a topic if I need to.’ (Participant 6, Female, Malawi)

Some reported a new focus on registrars in difficulty:

‘We have since developed an active program of identifying registrars in difficulty. We currently have two registrars we are supporting to overcome their current learning difficulties by offering the necessary support.’ (Participant 7, Male, Kenya)

## Discussion

A relatively brief course, to better equip FPs as clinical trainers in the workplace, was shown to have a positive effect on their self-assessed educational capability six weeks afterwards. FPs reported that they were better at ensuring safe and effective patient care through training, establishing and maintaining an environment for learning, teaching and facilitating learning, enhancing learning through assessment, and supporting and monitoring educational progress. FPs reported that they were better at giving feedback, more aware of different learning styles, more facilitative and less authoritarian in their educational approach, more reflective and critical of their educational capabilities and more aware of principles in assessment. Despite this, trainees did not report any noticeable change in the trainers’ capability after three months.

Not only does this research show evidence of impact on participants’ teaching practices but it also shows signs of changes to students’ learning and performance. This rises to the challenge recently posed by Spowart to ‘evidence’ the value of academic development endeavours for both political and practical purposes.^11^ They go on to argue that gauging impact requires reflection by the participants and that this should be integrated into courses. The daily and end-of-course reflections done by the participants and the way this research was conducted encouraged such reflections and may have sensitised the participants to apply what they had learnt on the course.

Despite these encouraging findings, the enhanced educational competencies of these FPs did not persist. In addition to the short course, the project also initiated a programme of formative assessment visits in the workplace that took place in the six months after the course.^12^ Senior FPs from each training programme were trained in a standardised approach to visiting FPs, assessing their ability as a clinical trainer, giving feedback and agreeing on a developmental plan. It is hoped that this process of formative assessment and development would become an ongoing activity in each programme. A special interest group for clinical trainers has also been created under the auspices of the South Africa Academy of Family Physicians (SAAFP).

A limitation of the before-and-after study design is that there was no control group and, theoretically, changes could have occurred for other reasons that were not controlled for. The researchers were not aware of any other educational initiatives that would have affected all the training programmes, and the changes that were reported were well aligned with the content and aims of the course. Self-reporting behaviour change could overestimate the effect of the course, and there could be some obsequiousness bias in the six-week assessment. Nevertheless, the measured changes were supported by the specific examples in the qualitative data.

The findings of this study support the value of continuing to offer this course in the South African context. Although a critical mass of FPs have already been trained, the intention should be for all existing and future clinical trainers to complete the course. In the long term, this might become a criterion for more formal accreditation as a clinical trainer. The South Africa Academy of Family Physicians has agreed to continue organising the course in South Africa.

The RCGP and Stellenbosch University have also agreed on a collaboration with Family Medicine Leadership, Education and Assessment Programme (FaM LEAP) to offer this course and the follow-up formative assessment visits to other countries in the region that are trying to develop postgraduate training in family medicine.

## Conclusion

This short course effectively enhanced the capability of FPs in the short term to embrace the role of clinical trainer in the workplace. The outcomes of this short course should be strengthened by ongoing formative assessment of the trainers to encourage their educational development. The short course should be offered to all South African FPs involved in clinical training and to other countries in the region with similar needs.
